# Multi-Sensor Registration in High-Precision CMM Based on a Composite Standard

**DOI:** 10.3390/s18041220

**Published:** 2018-04-16

**Authors:** Yan Zhao, Yiwen Wang, Xiuling Ye, Zhong Wang, Luhua Fu, Changjie Liu, Zhiwei Wang

**Affiliations:** 1 State Key Laboratory of Precision Measuring Technology and Instrument, School of Precision Instrument and Opto-Electronics Engineering, Tianjin University, Tianjin 300072, China; zy193707@tju.edu.cn (Y.Z.); wang_yw@tju.edu.cn (Y.W.); 2016202127@tju.edu.cn (X.Y.); wangzhong@tju.edu.cn (Z.W.); liuchangjie@tju.edu.cn (C.L.); 2 TZTEK Technology Co. Ltd., Suzhou 215153, China; wangzhiwei@tztek.com

**Keywords:** registration, multi-sensor, high-precision, composite standard

## Abstract

Registration is a critical step in multi-sensor dimensional measurement. As the accuracy of registration directly impacts the quality of final results, a reference sphere as a common standard is problematic in high-precision registration. In this paper, a novel method based on a composite standard is proposed to fuse the multiple heterogeneous sensors in high-precision coordinate measuring machines (CMMs), which will void the drawbacks of a reference sphere. The composite standard consists of a cone and cylinder, which share a same central axis. To ensure high precision in the submicron range, or better, the standard is manufactured by an ultra-precision machine. Three features of the composite standard are inspected by three sensors: a video camera (VC), a tactile probe (TP), and a chromatic confocal displacement sensor (CC). All features will concentrate on a common point through which the relation between the three sensors will be obtained. The errors of each measurement were analyzed theoretically, and simulations and real experiments were carried out to verify the composite standard. This study demonstrates that the proposed registration method is stable and that the standard has potential use for the registration of multiple sensors in high-precision dimensional measurement.

## 1. Introduction

Multi-sensor metrology is widely used in high-precision dimensional measurement, as workpieces become increasingly complicated and complex. A multi-sensor integrated system can meet the demands of holistic geometry and high precision, while also improving reliability and reducing uncertainty in the measurement data [1]. If holistic and usable information is desired by fusing results from different sensors, the registration of multiple sensors must be performed prior to any measurement. Registration is a basic and important step in multi-sensor dimensional measurement, and is used to match two or more datasets in all coordinate systems to obtain a common and unified format.

Registration methods can be classified into two major categories: registration based on material gauges and self-calibration [2]. With the former, coordinate systems are aligned with the help of common feature points. Bradley et al. [3] adopted a reference sphere to combine data from a contact probe and a laser scanner mounted on the z-arm of a coordinate measuring machine (CMM) into one common coordinate system. A set of three coordinate balls (3CB) was utilized to unify the laser scanner model and touch probe model by Jamshidi et al. in Reference [4]. Sładek et al. [5] calculated the transformation matrix from data of the reference sphere array, relying on full-field structured light optical scanning methods and data of contact measurement. Wei et al. [6] applied a thin ring gauge artifact to evaluate the probing errors of a multi-sensor CMM equipped with an imaging probe and a touch probe. Lu et al. [7] mounted a touch probe and a laser displacement sensor as close to each other as possible on the z-arm of a CMM. The two sensors were unified into one coordinate system by measuring a common sphere, so the laser displacement sensor could guide the touch probe. Self-calibration is used to register multiple coordinate systems of different sensors by measuring references in the system itself, instead of using standards provided on purpose. A multi-sensor CMM was designed by Shen et al. [8], in which a touch probe and 3D active vision system were integrated. A touch probe tip with small form error was utilized to make the registration of datasets. Besides the two major categories, Zhao et al. [9] present a new concept registration method with two material standards measured by different sensors, but this method is still not mature. In brief, there are many references about the registration of heterogeneous sensors; however, most of them have low precision or present other problems.

**Figure 1:**
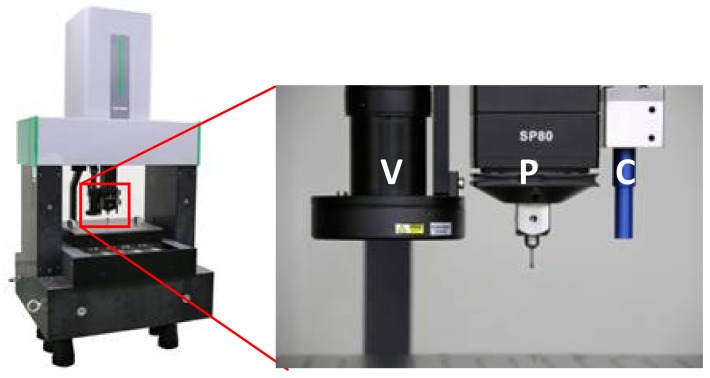
Multi-sensor high-precision coordinate measuring machine (CMM): V for video camera (VC), P for tactile probe (TP), C for chromatic confocal displacement sensor (CC).

In order to meet the demand of high accuracy in dimensional measurement, a high-precision CMM was constructed by a major partner, and a new method to register these different sensors by using a composite standard is proposed. The CMM is presented in Figure 1 with three different kinds of sensors: a video camera (VC), a tactile probe (TP), and a chromatic confocal displacement sensor (CC).

The composite standard is comprised of a cone and a cylinder which share a central axis. This standard is manufactured by an ultra-precision machine within nanometer order to ensure a more perfect standard. All features will concentrate on a common point through which the relation between the three sensors will be obtained. The common point can be the center of a circle or a sphere, or an intersection of two geometries, and so on.

Registration is based on the calibration of all sensors and adjustments of pose for each sensor, which are already done beforehand. Here, we focus on searching the origin or the common point in all sensor coordinate systems and the error propagation of the new composite standard.

This paper is structured as follows: in Section 2, we explore the problems that exist with the use of a reference sphere for high-precision measurement; in Section 3, we describe the registration principle; in Section 4, we present the mathematical description given to analyze the error propagation; in Section 5, the simulations and real experiments conducted to verify the composite standard are discussed; and Section 6 concludes the paper.

## 2. Problem Statement with a Reference Sphere

**Figure 2:**
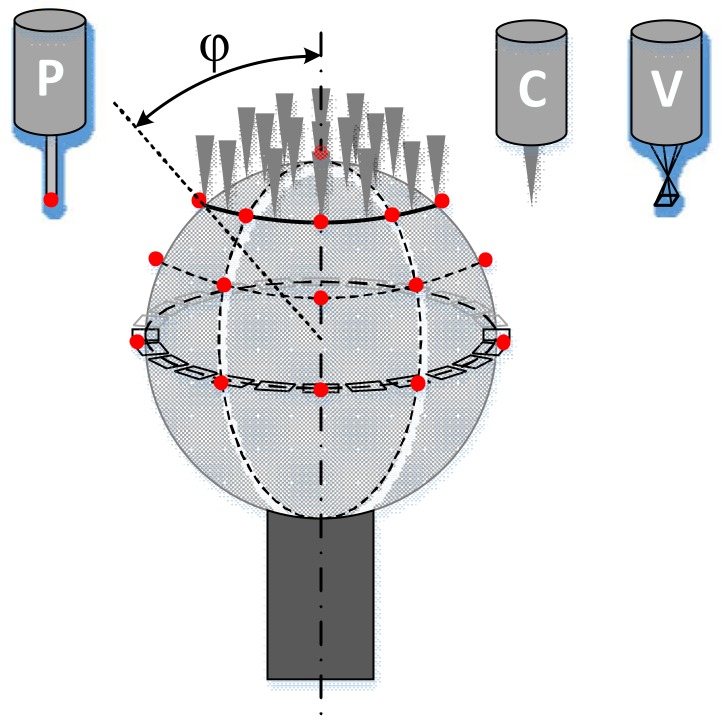
A reference sphere for multi-sensor registration.

Multi-sensor registration through the use of one or more reference spheres, as shown in Figure 2, is common in dimensional measurement. However, there are several problems in the registration of high-precision CMMs with the three sensors.

**Figure 3:**
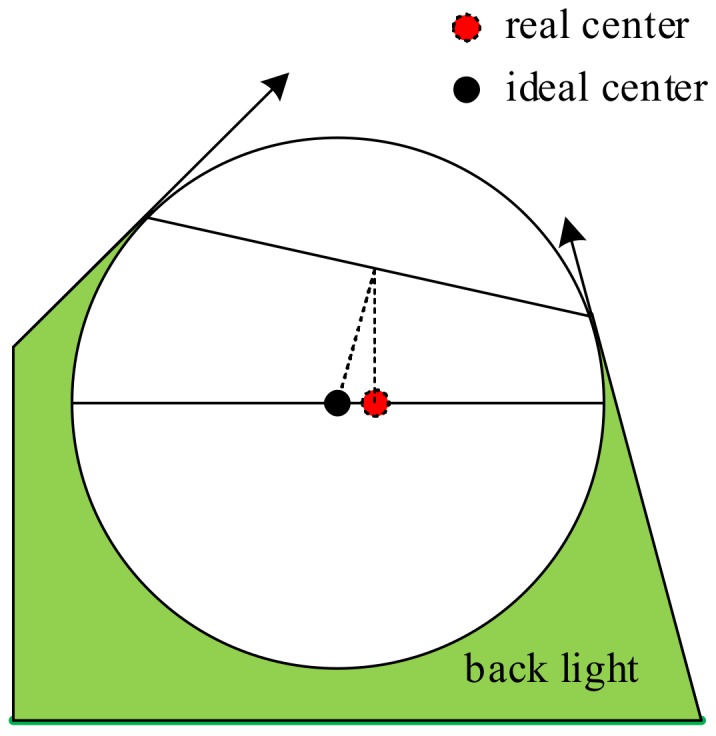
Light near the sphere.

For VCs, an imaging probe, a mask circle, or a jump edge in 2D is more suitable for the measuring principle. While the equatorial circle of a reference sphere is a gradually changing edge, the light near the edge of the equatorial circle will produce a special diffraction, which is different from that of a normal edge. More importantly, the interaction between the light source and the geometry of the sphere will lead to bias between the real center and the theoretical center, as shown in Figure 3.

**Figure 4:**
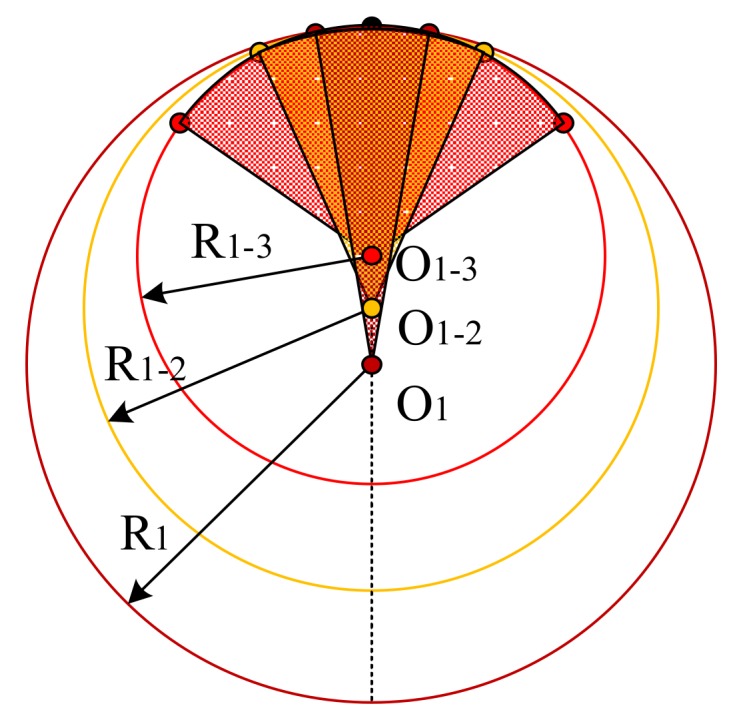
Centers of different coverages of the sphere.

For CCs, there is a limit on the angle between the optical axis of a CC and the measured plane, such that large angles will cause the sensor to fail. Thus, it is the low sample coverage of the measured points over the whole reference sphere that results in a poor performance on the circle center and radius. In actual measurements, it was found that the radius decreased as the coverage increased, while the coordinates on the z-axis were opposite, and the coordinates on the x-axis and y-axis were irregular. Figure 4 is a simplified description.

For the TPs, half of the whole sphere is touched with more than four sample points. In theory, the uncertainty should be approximately $\sqrt{2}$ times larger than that of the whole sphere.

**Figure 5:**
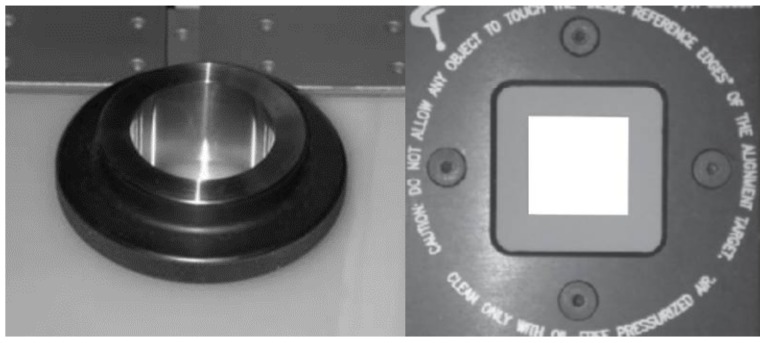
Ring gauge and rectangle.

A ring gauge is another well-known standard for the registration of a TP and a VC, and a sharp rectangle is used to unify a VC and a laser displacement sensor, as shown in Figure 5.

## 3. Multi-Sensor Registration with the New Composite Standard

**Figure 6:**
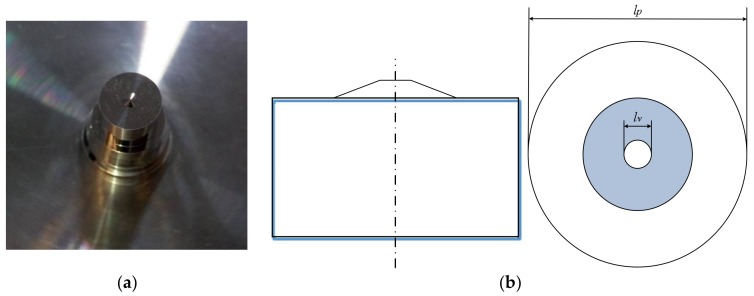
Geometries composite standard and computer-aided design (CAD) diagrams: (**a**) composite standard; (**b**) CAD diagrams of the standard.

Considering the drawbacks in using reference spheres in multi-sensor registration, a new standard was designed by us and manufactured by an ultra-precision machining center, where the form error of the standard could be as small as tens of nanometers. The standard consists of a stainless steel cone and a cylinder, as shown in Figure 6a. One of the advantages of the standard is the high-precision maturing machining technique, which yields a symmetrical object. The machining technique will ensure a specular surface and a precisely coaxial axis between the cone and the cylinder. In order to enforce the hardness of the standard for the TPs contacting it, a coating is used on the surface of the standard.

**Figure 7:**
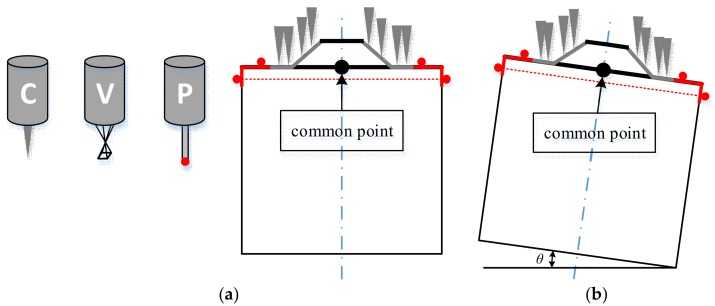
The standard measured by the three sensors: (**a**) diagram of the three sensors and ideal state of the standard; (**b**) inclined standard.

The center of the top plane of the cylinder is taken as the common point, also called the origin, as shown in Figure 7. To register the three sensors, all sensors should measure features to obtain the origin, then the relation between the three sensors will be clear. Here, the three sensors inspect three features of the composite standard respectively. The VC captures the top circle of the cone, which is a sharp feature suitable for VCs. The TP contacts the cylinder, both on its top plane and the generatrix for the intersection point of the top plane and the symmetry axis of the cylinder. The CC scans the generatrix of the cone and the top plane of the cylinder to get the center of the bottom circle of the cone. In fact, the center of the top circle and the center of the bottom circle of the cone are not the same, but the deviation can be determined using the known distance between them and the normal vector of the top plane, so that all the sensors can be traced to the origin.

### 3.1. Center of the Top Circle of the Cone with the VC

As the VC is always equipped with an orthogonal lens for high-precision measurement and the surface of the standard is specular, only the normal plane of the surface perpendicular to the optic axis of the VC can inter the lens, and the VC can capture circles of the cone by using top coaxial light. We can fit these circles using the least squares method. Let R be the radius, and $X_{cv}$, $Y_{cv}$, and $Z_{cv}$ be the *x*, *y,* and *z* coordinates of the circle center ($P_{vc}$).

(1) $$\begin{array}{ll} R^{2} & ={\left(x-X_{cv}\right)}^{2}+{\left(y-Y_{cv}\right)}^{2}\\ & =x^{2}-2X_{cv}x+X_{cv}^{2}+y^{2}-2Y_{cv}y+Y_{cv}^{2} \end{array}$$

Let: $e=-2X_{cv},\;f=-2Y_{cv}\;,s=X_{cv}^{2}+Y_{cv}^{2}-R^{2}$, there is:

(2) $$x^{2}+y^{2}+ex+fy+s=0$$

Equation (2) is more suitable for least squares fitting compared to Equation (1). Additionally, $Z_{cv}$ can be obtained by focusing the edge of the measured circle.

Since R is useless for the position of the VC, only the center $P_{vc}=\left(X_{cv},X_{cv},Z_{cv}\right)$ is considered.

### 3.2. Center of the Top Plane of the Cylinder

In the real case, the standard will never ideally be fixed on the working stage—an inclined angle always exists, as shown in Figure 7. To void this, the intersection point of the top plane and the symmetry axis of the cylinder is used to precisely locate the center of the top plane. As the normal vector of the top plane and the symmetry axis are the same, only one point on the symmetry axis should be determined. This point is obtained by calculating the center of a certain circle of the cylinder using Equation (2).

The TP detects the top plane of the cylinder, except for the area of the cone to avoid outlier points. Let *A*, *B*, *C*, and *D* be the parameters of the plane equation, there is:

(3) Ax+By+Cz+D=0(C≠0).

Equation (3) can also be written as follows, where $a=\frac{A}{C}\;,b=\frac{B}{C},d=\frac{D}{C}$:

(4) ax+by+z+d=0.

In order to get a solution for Equation (4), the least squares method will also be used.

The symmetry line can be expressed as Equation (5):

(5) $$\left\{ \begin{array}{l} x=x_{0}+a\left(z-z_{0}\right)\\ y=y_{0}+b\left(z-z_{0}\right) \end{array}\right.,$$

where $\left(x_{0},y_{0},z_{0}\right)$ is the point on the symmetry axis of the cylinder, and (a,b,1) is the special normal vector.

The intersection point is Equation (6):

(6) $$\left\{ \begin{array}{l} Z_{cp}=\frac{-ax_{0}-by_{0}+a^{2}z_{0}+b^{2}z_{0}-d}{1+a^{2}+b^{2}}\\ X_{cp}=\frac{\left(1+b^{2}\right)x_{0}-aby_{0}-az_{0}-ad}{1+a^{2}+b^{2}}\\ Y_{cp}=\frac{-abx_{0}+\left(1+a^{2}\right)y_{0}-bz_{0}-bd}{1+a^{2}+b^{2}} \end{array}\right..$$

### 3.3. Center of the Intersection Circle of the Cone and the Top Plane of the Cylinder

The CC will scan the cone and the top plane of the cylinder as parts with grey triangles, as shown in Figure 7. Both equations of the cone and the plane will be obtained when there is no inclined angle for the standard on the working stage. The center of the bottom circle of the cone, as well as the center of the intersection circle of the cone and the top plane of the cylinder, will be obtained easily, which represents a symbolic point for the CC. In the case of an inclined angle, an optimization method is applied in order to avoid the complexity of the computation. The inclined angle θ is very small, but cannot be ignored without any adjustment. The least-squares orthogonal distances fitting method in Reference [10] is adopted to obtain the center of the intersection circle.

Suppose that q parameters a are assumed to be related to the p(≥q) measurements **X** according to

(7) X=F(a)+e,

where **F** represents some nonlinear continuously differentiable observation functions of a, and e denotes errors with zero mean. The Gauss–Newton iteration with initial parameters vector $a_{0}$ and step-size parameter λ was chosen to solve the parameters.

(8) $${\frac{\partial F}{\partial a}|}_{a_{k}}=X-F\left(a_{k}\right)$$

(9) $$a_{k+1}=a_{k}+\lambda \Delta a$$

At first, transform the standard with an inclined angle to an ideal state without an inclined angle:

(10) $$x=R\left(X-X_{c}\right).$$

$x={\left(x,y,z\right)}^{T}$ is the point in an ideal coordinate system, $X={\left(X,Y,Z\right)}^{T}$ is the point in a real coordinate system, and $X_{c}={\left(X_{cc},Y_{cc},Z_{cc}\right)}^{T}$ is the center of the intersection circle of the cone and is also set to be the origin point.

(11) $$\begin{array}{l} R=Rx\cdot Ry\cdot Rz\\ =\left[\begin{array}{ccc} \cos\left[\beta \right]\cos\left[\gamma \right] & \cos\left[\beta \right]\sin\left[\gamma \right] & \sin\left[\beta \right]\\ \cos\left[\gamma \right]\sin\left[\alpha \right]\sin\left[\beta \right]-\cos\left[\alpha \right]\sin\left[\gamma \right] & \cos\left[\alpha \right]\cos\left[\gamma \right]+\sin\left[\alpha \right]\sin\left[\beta \right]\sin\left[\gamma \right] & -\cos\left[\beta \right]\sin\left[\alpha \right]\\ -\cos\left[\alpha \right]\cos\left[\gamma \right]\sin\left[\beta \right]-\sin\left[\alpha \right]\sin\left[\gamma \right] & \cos\left[\gamma \right]\sin\left[\alpha \right]-\cos\left[\alpha \right]\sin\left[\beta \right]\sin\left[\gamma \right] & \cos\left[\alpha \right]\cos\left[\beta \right] \end{array}\right] \end{array},$$

where (α,β,γ) are the angles rotated along/around *x*, *y*, and *z* axes.

After the transformation, we will get an ideal cone and an ideal plane Equation (12):

(12) $$\left\{ \begin{array}{l} cone:z=k\sqrt{x^{2}+y^{2}}+l\\ line:z=0 \end{array}\right..$$

For a given point $\left(x_{i},y_{i},z_{i}\right)$, the tangent plane at the orthogonal contacting point (x,y,z) on the cone or plane and the connecting line of the two points are perpendicular to each other. The nearest corresponding point $X_{i}^{\prime }$ ($X_{c}^{\prime }$ for the cone and $X_{p}^{\prime }$ for the plane) will be known according to the geometric relationship between the given point and the cone or the plane.

Let parameters vector $a={\left(\alpha ,\beta ,\gamma ,X_{cc},Y_{cc},Z_{cc},k,l\right)}^{T}$. The Jacobian matrix **J** is Equation (13):

(13) $$J_{X_{i}^{\prime },a}={\frac{\partial X}{\partial a}|}_{X=X_{i}^{\prime }}={\left(R^{-1}\frac{\partial x}{\partial a}+\frac{\partial R^{-1}}{\partial a}x+\frac{\partial X_{c}}{\partial a}\right)|}_{X=X_{i}^{\prime }}$$

$\frac{\partial x}{\partial a}$ can be deduced with the following Equations (14) and (15).

(14) $$\frac{\partial x_{i}}{\partial a}=\frac{\partial R}{\partial a}\left(X_{i}-X_{c}\right)-R\frac{\partial X_{c}}{\partial a}\;,$$

(15) $$\left(\begin{array}{l} \frac{\partial f_{1}}{\partial a}\\ \frac{\partial f_{2}}{\partial a}\\ \frac{\partial f_{3}}{\partial a} \end{array}\right)=\left(\begin{array}{c} \frac{\partial f_{1}}{\partial \alpha }\;\frac{\partial f_{1}}{\partial \beta }\;\frac{\partial f_{1}}{\partial \gamma }\;\frac{\partial f_{1}}{\partial X_{cc}}\;\frac{\partial f_{1}}{\partial Y_{cc}}\;\frac{\partial f_{1}}{\partial Z_{cc}}\;\frac{\partial f_{1}}{\partial k}\;\frac{\partial f_{1}}{\partial l}\\ \frac{\partial f_{2}}{\partial \alpha }\;\frac{\partial f_{2}}{\partial \beta }\;\frac{\partial f_{2}}{\partial \gamma }\;\frac{\partial f_{2}}{\partial X_{cc}}\;\frac{\partial f_{2}}{\partial Y_{cc}}\;\frac{\partial f_{2}}{\partial Z_{cc}}\;\frac{\partial f_{2}}{\partial k}\;\frac{\partial f_{2}}{\partial l}\\ \frac{\partial f_{3}}{\partial \alpha }\;\frac{\partial f_{3}}{\partial \beta }\;\frac{\partial f_{3}}{\partial \gamma }\;\frac{\partial f_{3}}{\partial X_{cc}}\;\frac{\partial f_{3}}{\partial Y_{cc}}\;\frac{\partial f_{3}}{\partial Z_{cc}}\;\frac{\partial f_{3}}{\partial k}\;\frac{\partial f_{3}}{\partial l} \end{array}\right)=0\;,$$

where $f_{i}$ involves $f_{ci}$ and $f_{pi}$, the intersection points of the cone and the plane are in Equations (16) and (17):

(16) $$Cone:\{\begin{array}{c} f_{c1}\left(x,y,z\right)=z+k\sqrt{x^{2}+y^{2}}-l\\ f_{c2}\left(x,y,z\right)=kx\left(z_{i}-z\right)-\left(x_{i}-x\right)\sqrt{x^{2}+y^{2}}\\ f_{c3}\left(x,y,z\right)=ky\left(z_{i}-z\right)-\left(y_{i}-y\right)\sqrt{x^{2}+y^{2}} \end{array},$$

(17) $$Plane:\{\begin{array}{c} f_{p1}\left(x,y,z\right)=z\\ f_{p2}\left(x,y,z\right)=x_{i}-x\\ f_{p3}\left(x,y,z\right)=y_{i}-y \end{array}\;.$$

The orthogonal error distances vector will be:

(18) $$X_{i}^{\prime \prime }=X_{i}-X_{i}^{\prime }\;.$$

With the Jacobian matrix $J_{X_{i}^{\prime },a}$ in Equation (13) and the error distances vector $X_{i}^{\prime \prime }$ in Equation (18), at each point $X_{i}^{\prime }$, $p\left(=3n=3\times \left(n_{c}+n_{p}\right)\right)$ linear equations for the m given 3D points of both the cone and the plane can be constructed in Equation (19).

(19) $$\left(\begin{array}{c} J_{X_{1}^{\prime },\alpha }\;J_{X_{1}^{\prime },\beta }\;J_{X_{1}^{\prime },\gamma }\;J_{X_{1}^{\prime },X_{cc}}\;J_{X_{1}^{\prime },Y_{cc}}\;J_{X_{1}^{\prime },Z_{cc}}\;J_{X_{1}^{\prime },k}\;J_{X_{1}^{\prime },l}\\ J_{Y_{1}^{\prime },\alpha }\;J_{Y_{1}^{\prime },\beta }\;J_{Y_{1}^{\prime },\gamma }\;J_{Y_{1}^{\prime },X_{cc}}\;J_{Y_{1}^{\prime },Y_{cc}}\;J_{Y_{1}^{\prime },Z_{cc}}\;J_{Y_{1}^{\prime },k}\;J_{Y_{1}^{\prime },l}\\ J_{Z_{1}^{\prime },\alpha }\;J_{Z_{1}^{\prime },\beta }\;J_{Z_{1}^{\prime },\gamma }\;J_{Z_{1}^{\prime },X_{cc}}\;J_{Z_{1}^{\prime },Y_{cc}}\;J_{Z_{1}^{\prime },Z_{cc}}\;J_{Z_{1}^{\prime },k}\;J_{Z_{1}^{\prime },l}\\ \vdots \;\vdots \;\vdots \;\vdots \;\vdots \;\vdots \;\vdots \;\\ \vdots \;\vdots \;\vdots \;\vdots \;\vdots \;\vdots \;\vdots \;\\ J_{X_{n}^{\prime },\alpha }\;J_{X_{n}^{\prime },\beta }\;J_{X_{n}^{\prime },\gamma }\;J_{X_{n}^{\prime },X_{cc}}\;J_{X_{n}^{\prime },Y_{cc}}\;J_{X_{n}^{\prime },Z_{cc}}\;J_{X_{n}^{\prime },k}\;J_{X_{n}^{\prime },l}\\ J_{Y_{n}^{\prime },\alpha }\;J_{Y_{n}^{\prime },\beta }\;J_{Y_{n}^{\prime },\gamma }\;J_{Y_{n}^{\prime },X_{cc}}\;J_{Y_{n}^{\prime },Y_{cc}}\;J_{Y_{n}^{\prime },Z_{cc}}\;J_{Y_{n}^{\prime },k}\;J_{Y_{n}^{\prime },l}\\ J_{Z_{n}^{\prime },\alpha }\;J_{Z_{n}^{\prime },\beta }\;J_{Z_{n}^{\prime },\gamma }\;J_{Z_{n}^{\prime },X_{cc}}\;J_{Z_{n}^{\prime },Y_{cc}}\;J_{Z_{n}^{\prime },Z_{cc}}\;J_{Z_{n}^{\prime },k}\;J_{Z_{n}^{\prime },l} \end{array}\right)\left(\begin{array}{c} \begin{array}{c} \begin{array}{c} \Delta \alpha \\ \Delta \beta \end{array}\\ \begin{array}{c} \Delta \gamma \\ \Delta X_{cc} \end{array} \end{array}\\ \begin{array}{c} \begin{array}{c} \Delta Y_{cc}\\ \Delta Z_{cc} \end{array}\\ \begin{array}{c} \Delta k\\ \Delta l \end{array} \end{array} \end{array}\right)=\left(\begin{array}{c} \begin{array}{c} \begin{array}{c} X_{1}^{\prime \prime }\\ Y_{1}^{\prime \prime } \end{array}\\ \begin{array}{c} Z_{1}^{\prime \prime }\\ \vdots \end{array} \end{array}\\ \begin{array}{c} \begin{array}{c} \vdots \\ X_{n}^{\prime \prime } \end{array}\\ \begin{array}{c} Y_{n}^{\prime \prime }\\ Z_{n}^{\prime \prime } \end{array} \end{array} \end{array}\right)$$

$\left(X_{cc},Y_{cc},Z_{cc}\right)$ is the final center for the CC. The initial parameters vector can be rough data with a simple calculation.

Whereas the standard is a revolving geometry with a high precision, only the center is under consideration, so one of the three angles can be eliminated for simplicity.

According to Equations (4) and (19), we find the common point or the origin in both the TP and the CC coordinate systems. Equation (2) also provides the coordinates of the point higher than the origin with a constant known distance, so the relation of position between the three sensors will be determined.

## 4. Error Propagation

The errors of each measurement were analyzed theoretically, and the mathematical description is given to analyze the error propagation.

### 4.1. Errors of Circle Center for the VC

**Figure 8:**
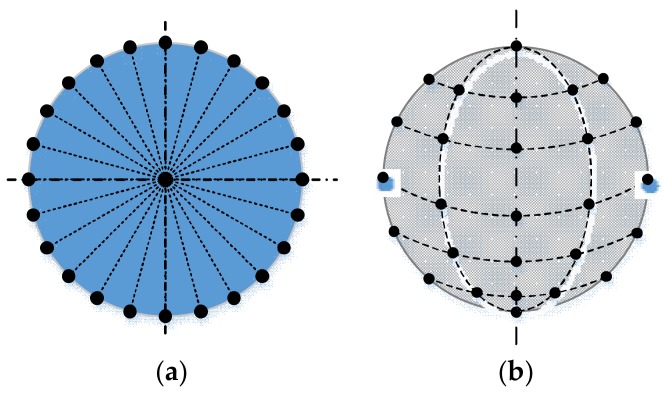
Sample strategy of a circle and a sphere: (**a**) sample points on a circle; (**b**) sample points on a sphere.

The whole circle is assumed to be sampled uniformly, as shown in Figure 8a. While it is difficult to be strictly uniform for the sample points on a sphere, the sphere is sampled uniformly in longitude and latitude, as shown in Figure 8b.

For a circle according to Reference [11], there are errors of both *x* and *y* coordinates of the center:

(20) $$\{\begin{array}{c} \delta_{O}=\frac{2∆}{\sqrt{N}}\\ \delta_{x}=∆\sqrt{\frac{2}{N}}\\ \delta_{y}=∆\sqrt{\frac{2}{N}} \end{array}.$$

For a sphere, the center errors are:

(21) $$\{\begin{array}{c} \delta_{x}=∆\sqrt{\frac{2}{4+\left(K-2\right)N_{K}}}=∆\sqrt{\frac{2}{N+2}}\\ \delta_{y}=∆\sqrt{\frac{2}{4+\left(K-2\right)N_{K}}}=∆\sqrt{\frac{2}{N+2}}\\ \delta_{z}=\frac{2∆}{\sqrt{\left(K-2\right)N_{K}}}=\frac{2∆}{\sqrt{N-2}} \end{array}\;,$$

with $N=2+\left(K-2\right)N_{K}$, where *K* is the number of the layer of the sample circle with a certain quantity $N_{K}$ of sample points, *N* is the total number of sample points, and ∆ is the final precision of the machine with sensors.

From Equations (20) and (21), it is clear that the center precision is mainly related to the number of sample points and the precision of the sensor; as the number grows, the precision of the circle center will increase.

### 4.2. Errors of Intersection Circle Center for the CC

After a successful termination of the iteration procedure, the information regarding the quality of the fitting parameters will be provided [8]. The parameter covariance matrix in Equation (22):

(22) $$\mathrm{cov}\left(a\right)={\left(J^{T}J\right)}^{-1}={\left(VWU^{T}UWV^{T}\right)}^{-1}=VW^{-2}V^{T}$$

with $U^{T}U\;=\;V^{T}V\;=\;I$, $W=\left[\mathrm{diag}\left(w_{1},\ldots w_{q}\right)\right]$, and the covariance is written in Equation (23):

(23) $$\mathrm{cov}\left(a_{j},a_{k}\right)=\overset{q}{\underset{i=1}{\sum }}\left(\frac{V_{ji}V_{ki}}{w_{i}^{2}}\right)\;j=1,\ldots ,q,\;k=1,\ldots ,q,$$

so the variance of the estimated parameters is:

(24) $$\sigma^{2}\left(a_{j}\right)=\frac{\sigma_{0}^{2}}{p-q}cov\left(a_{j},a_{j}\right),\;j=1,\ldots ,q,$$

where $\sigma_{0}^{2}={\left[X-F\left(\hat{a}\right)\right]}^{T}\left[X-F\left(\hat{a}\right)\right]$.

The correlation coefficients are:

(25) $$\rho \left(a_{j},a_{k}\right)=\frac{cov\left(a_{j},a_{k}\right)}{\sqrt{cov\left(a_{j},a_{j}\right)cov\left(a_{k},a_{k}\right)}}\;.$$

According to Equations (24) and (25), errors of all coordinates of the center will be known and the correlation between them will also be obtained.

### 4.3. Errors of the Top Plane Center of the Cylinder 

For a linear least squares fitting (e.g., a line or a plane), errors of all parameters will be obtained by Equation (26):

(26) $$\begin{array}{ll} DX & =E\left(X-EX\right)\left(X-EX\right)\\ & ={\left(A^{T}A\right)}^{-1}A^{T}E\left(L-AX\right){\left(L-AX\right)}^{T}{\left[{\left(A^{T}A\right)}^{-1}A^{T}\right]}^{T}\\ & ={\left(A^{T}A\right)}^{-1}A^{T}DLA{\left(A^{T}A\right)}^{-1}\\ & ={\left(A^{T}A\right)}^{-1}A^{T}\sigma^{2}I{\left(A^{T}A\right)}^{-1}\\ & ={\left(A^{T}A\right)}^{-1}\sigma^{2} \end{array}$$

with ${\left(A^{T}A\right)}^{-1}=\left[\begin{array}{cc} \begin{array}{cc} d_{11} & d_{12}\\ d_{21} & d_{22} \end{array} & \begin{array}{cc} \cdots & d_{1n}\\ \cdots & d_{2n} \end{array}\\ \begin{array}{cc} \vdots & \vdots \\ d_{n1} & d_{n2} \end{array} & \begin{array}{cc} \vdots & \vdots \\ \ldots & d_{nn} \end{array} \end{array}\right]$, $\sigma^{2}=\frac{{\left(L-AX\right)}^{T}\left(L-AX\right)}{n},$ so the covariance of the estimated parameters is $\sigma_{ij}=\sigma \sqrt{d_{ij}}$.

According to Equation (6), the error of the *z* coordinate can be written as:

(27) $$\begin{array}{l} \Delta z^{2}={\left(\frac{\delta z}{\delta a}\right)}^{2}\Delta a^{2}+{\left(\frac{\delta z}{\delta b}\right)}^{2}\Delta b^{2}+{\left(\frac{\delta z}{\delta d}\right)}^{2}\Delta d^{2}+{\left(\frac{\delta z}{\delta x_{0}}\right)}^{2}\Delta x_{0}^{2}+{\left(\frac{\delta z}{\delta y_{0}}\right)}^{2}\Delta y_{0}^{2}+{\left(\frac{\delta z}{\delta z_{0}}\right)}^{2}\Delta z_{0}^{2}\\ +2\rho_{ab}\left(\frac{\delta z}{\delta a}\right)\left(\frac{\delta z}{\delta b}\right)\Delta a\Delta b+2\rho_{ad}\left(\frac{\delta z}{\delta a}\right)\left(\frac{\delta z}{\delta d}\right)\Delta a\Delta d+2\rho_{bd}\left(\frac{\delta y}{\delta b}\right)\left(\frac{\delta y}{\delta d}\right)\Delta b\Delta d\\ +2\rho_{x_{0}y_{0}}\left(\frac{\delta z}{\delta x_{0}}\right)\left(\frac{\delta z}{\delta y_{0}}\right)\Delta x_{0}\Delta y_{0}+2\rho_{x_{0}z_{0}}\left(\frac{\delta z}{\delta x_{0}}\right)\left(\frac{\delta z}{\delta z_{0}}\right)\Delta x_{0}\Delta z_{0}+2\rho_{y_{0}z_{0}}\left(\frac{\delta y}{\delta y_{0}}\right)\left(\frac{\delta y}{\delta z_{0}}\right)\Delta y_{0}\Delta z_{0} \end{array}.$$

If the intersection point is set to be the origin, Equation (27) can be simplified as:

(28) $$\begin{array}{l} \Delta z^{2}={\left(\frac{\delta z}{\delta a}\right)}^{2}\Delta a^{2}+{\left(\frac{\delta z}{\delta b}\right)}^{2}\Delta b^{2}+{\left(\frac{\delta z}{\delta d}\right)}^{2}\Delta d^{2}+{\left(\frac{\delta z}{\delta x_{0}}\right)}^{2}\Delta x_{0}^{2}+{\left(\frac{\delta z}{\delta y_{0}}\right)}^{2}\Delta y_{0}^{2}+{\left(\frac{\delta z}{\delta z_{0}}\right)}^{2}\Delta z_{0}^{2}\\ +2\rho_{ab}\left(\frac{\delta z}{\delta a}\right)\left(\frac{\delta z}{\delta b}\right)\Delta a\Delta b+2\rho_{ad}\left(\frac{\delta z}{\delta a}\right)\left(\frac{\delta z}{\delta d}\right)\Delta a\Delta d+2\rho_{bd}\left(\frac{\delta y}{\delta b}\right)\left(\frac{\delta y}{\delta d}\right)\Delta b\Delta d \end{array}.$$

The errors of *x* coordinate and *y* coordinate can be written as:

(29) $$\begin{array}{l} \Delta x^{2}={\left(\frac{\delta x}{\delta a}\right)}^{2}\Delta a^{2}+{\left(\frac{\delta x}{\delta b}\right)}^{2}\Delta b^{2}+{\left(\frac{\delta x}{\delta d}\right)}^{2}\Delta d^{2}+{\left(\frac{\delta x}{\delta x_{0}}\right)}^{2}\Delta x_{0}^{2}+{\left(\frac{\delta x}{\delta y_{0}}\right)}^{2}\Delta y_{0}^{2}+{\left(\frac{\delta x}{\delta z_{0}}\right)}^{2}\Delta z_{0}^{2}\\ +2\rho_{ab}\left(\frac{\delta x}{\delta a}\right)\left(\frac{\delta x}{\delta b}\right)\Delta a\Delta b+2\rho_{ad}\left(\frac{\delta x}{\delta a}\right)\left(\frac{\delta x}{\delta d}\right)\Delta a\Delta d+2\rho_{bd}\left(\frac{\delta x}{\delta b}\right)\left(\frac{\delta x}{\delta d}\right)\Delta b\Delta d \end{array},$$

(30) $$\begin{array}{l} \Delta y^{2}={\left(\frac{\delta y}{\delta a}\right)}^{2}\Delta a^{2}+{\left(\frac{\delta y}{\delta b}\right)}^{2}\Delta b^{2}+{\left(\frac{\delta y}{\delta d}\right)}^{2}\Delta d^{2}+{\left(\frac{\delta y}{\delta x_{0}}\right)}^{2}\Delta x_{0}^{2}+{\left(\frac{\delta y}{\delta y_{0}}\right)}^{2}\Delta y_{0}^{2}+{\left(\frac{\delta y}{\delta z_{0}}\right)}^{2}\Delta z_{0}^{2}\\ +2\rho_{ab}\left(\frac{\delta y}{\delta a}\right)\left(\frac{\delta y}{\delta b}\right)\Delta a\Delta b+2\rho_{ad}\left(\frac{\delta y}{\delta a}\right)\left(\frac{\delta y}{\delta d}\right)\Delta a\Delta d+2\rho_{bd}\left(\frac{\delta y}{\delta b}\right)\left(\frac{\delta y}{\delta d}\right)\Delta b\Delta d \end{array}.$$

Among Equations (28)–(30), errors of parameters and the correlation coefficients are known from Equation (26). As a result, errors of the intersection center will be obtained. Errors of the offsets between different sensors can then be calculated simply with combinations of the errors above.

## 5. Experiments

In order to verify the registration method with the composite standard, both simulations and real experiments were carried out. Standard deviation (SD) was used to denote an uncertainty of an element.

### 5.1. Simulations

To observe the registration precision with the composite standard, 200 simulations were conducted in total for each sensor. The mean of each coordinate and the SD are listed in tables.

#### 5.1.1. Simulations of Circles Captured by the VC

At first, an inclined circle in 3D was constructed with a normal vector [0.03, 0.04, 1]. The radius was 0.1 mm, the distance from the circle center to the origin was 0.25 mm in height, and the origin was [80, 50, 70] mm in the VC’s coordinate system. The noise with standard deviation [0.001, 0.001, 0.005] mm was added to the circle data.

**Figure 9:**
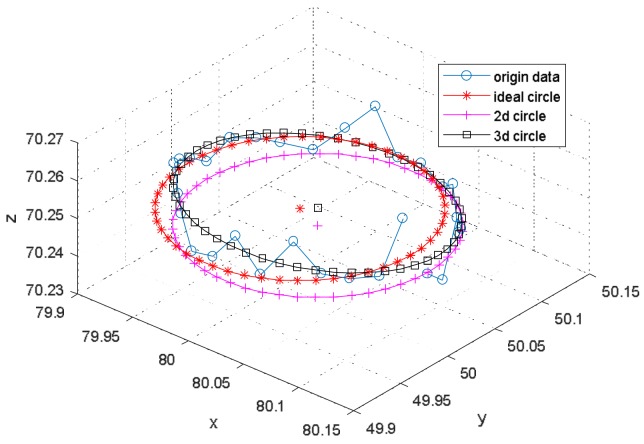
Simulated circle captured by the VC.

An ellipse fitted with a circle fitting function in 2D will still get a precise center, just as with the center of the circle shown in Figure 9 and in Table 1, when there is an inclined circle for the VC. The error of the *z* coordinate of the fitted circle in 2D in Table 1 will rely on the number of repetitions and the accuracy in the *z*-axis direction.

#### 5.1.2. Simulations of the Intersection Point for the TP

The symmetry axis of the cylinder was also set as [0.03, 0.04, 1]. The origin was [100, 60, 50] mm in the TP coordinate system, and the noise with SD was [0.001, 0.001, 0.001] mm. One point on the symmetry axis of the cylinder was obtained by fitting the circle, precisely the ellipse, with a cross section at height *z* = 49 mm.

**Figure 10:**
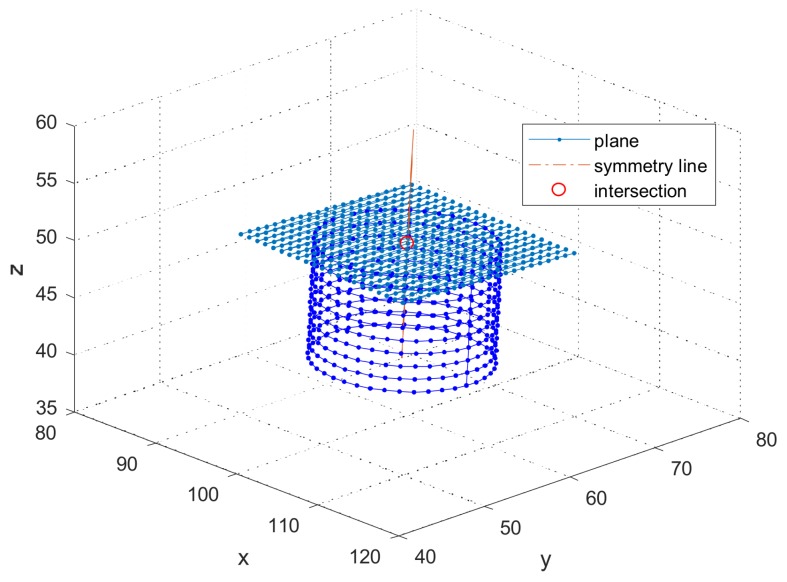
Simulated plane and cylinder touched by the TP.

Figure 10 shows that the simulated plane and the simulated cylinder symmetry axis intersect at the origin. In Table 2, in spite of the same noise in three directions, the error of the *z* coordinate of the origin is much smaller than others; this is mainly affected by the sample points of the plane.

#### 5.1.3. Simulations of the Intersection of a Cone and a Plane for the CCs

[0.03, 0.04, 1] was still the normal vector of the symmetry axis. The origin in the CC coordinate system was set as [100, 75, 60] mm. The standard deviation of the noise was [0.001, 0.001, 0.001] mm.

**Figure 11:**
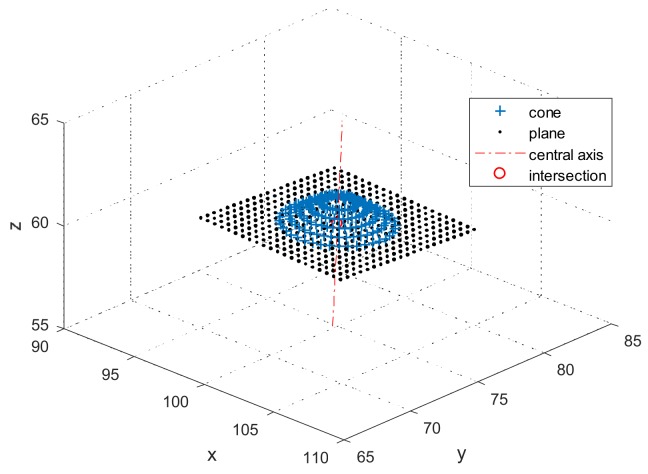
Simulated plane and cone scanned by the CC.

The simulated data are shown in Figure 11, and the results in Table 3. In Table 3, the SDs are larger than those in Table 1 and Table 2 with multiple simulations. The reason may be that it is difficult to use the optimization method to obtain precise results without adequate constraints. The SDs are still superior to that of the noise added in the sample points. More importantly, there is no systematic error in the results of the CC, compared with a reference sphere. If more points are measured, good results will be obtained.

#### 5.1.4. Relative Position of All Sensors

In Table 1, it has a center 0.25 mm higher than the origin, so it is necessary to compute the origin in the VC coordinate system. As the normal vector is [0.03, 0.04, 1], the origin point in the VC coordinate system will be [80, 50, 70]. According to Table 2 and Table 3, the offsets from the VC to the TP are [−20.000, −10.000, 20.000] mm, with a SD [0.3439, 0.3355, 0.8211] µm, and the offsets from the CC to the TP are [0.000, 17.000, 10.000] mm, with a SD [0.7433, 0.6462, 0.6519] µm.

In theory, from these simulations, the composite standard can be used to conduct the registration of multiple sensors in a high CMM, and a stable result will be achieved.

### 5.2. Real Experiments

A new machine made by our major partner is shown in Figure 1. There are three sensors: a VC, a TP, and a CC, which are mounted on the z-arm of the CMM with constant positions.

The maximum permissible errors (MPEs) for each sensor are E3 = (2.2 + L/400) μm in 3D space, and E1 < (1.0 + L/600) μm for 1D. The field of view (FOV) of the VC is 0.4 mm × 0.3 mm, the resolution is 2448 × 2048 pixels, and the magnification is 20×. The MPE of the TP is 0.5 + L/1000 μm, with a resolution 0.02 μm. The working distance of the CC is 4500 μm, and the measuring range is 300 μm with an MPE of 0.1 μm.

**Figure 12:**
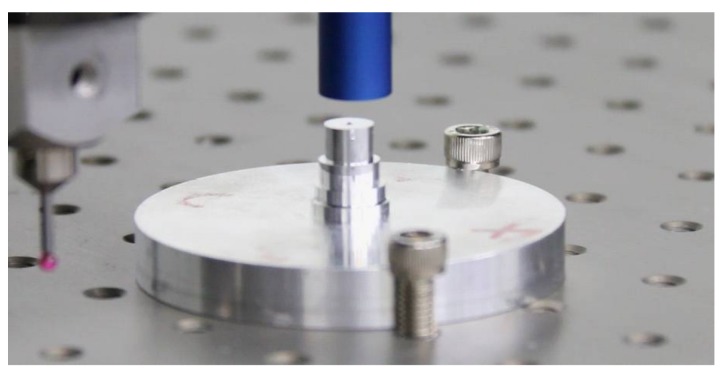
The standard measured by multiple sensors.

**Figure 13:**
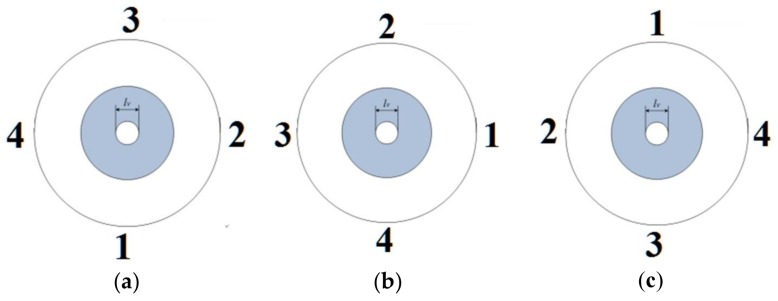
The rotated standard: (**a**) position 1; (**b**) position 2; (**c**) position 3.

The standard in Figure 12 was measured by all three sensors, five times in each position, and the standard was rotated three times with an interval of approximately 90 degrees on the working stage, as shown in Figure 13.

Registration results are listed in Table 4 for the offsets from the VC to the TP, and in Table 5 for the offsets from the CC to the TP.

From Table 4, we can see that from the offsets in *x* and *y* directions, PV-dx and PV-dy were liable to the rotation of the standard. This implies that the top circle of the cone and the symmetry axis may be not coaxial. The reason could be that the light used by the VC was not ideal, compared to the results in Table 5.

In Table 5, from the offsets in *x* and *y* directions, the PC-dx and PC-dy between the CC and the TP were more stable. This indicates a large error in the VC measurements of the top circle of the cone.

In fact, the value in the *z* direction of the VC is out of consideration for high-precision measurements. This is because the VC is mainly applied to obtain the dimensions in 2D.

The offsets between the CC and the TP calculated by a basic computation are listed in Table 6, and the results were similar. However, PC-dx and PC-dy with a Newton iteration method were better than with a basic computation, while PC-dz was just the opposite.

For single measurement with each sensor, the error can be calculated by equations in Section 4. These equations or functions will assess whether the feature is suitable to be used to represent the origin or the common point.

A sphere was also adopted to register the three sensors, and the results are listed in Table 7. Compared to the results in Table 4, Table 5 and Table 6, the results of the sphere seemed to be more stable. This is because the SDs in Table 7 were deduced from multiple measurements at one location, while the composite standard was placed at three different locations. The offset PV-dx (absolute value) in Table 7 was slightly smaller, which may be caused by the error shown in Figure 3. Regarding the offsets between the TP and the CC, the offset PC-dz (absolute value) was about 1–3 µm larger, which could be the error in Figure 4.

According to these tables, we can conclude that the offsets obtained by both standards were similar; the deviations of all the offsets conformed to the final MPE ($\mathrm{Es}=\;\sqrt{2}\times E3$) of the CMM in 3D; the new composite standard could be used to register the three sensors in the CMM; additionally, there were some inconsistent offsets compared to that of a sphere, which could be caused by the unsuitable features measured by sensors.

## 6. Conclusions

In this paper, a registration method based on a composite standard was presented. By measuring different features of the standard, the three sensors could obtain the common point or the origin in each sensor coordinate system, after which the registration was achieved according to the relation between them. The fitting errors of each feature were analyzed theoretically with mathematical descriptions, and both synthetic data and real experiments were carried out to validate the feasibility and the stability of the method based on the composite standard. The results show that the registration method was able to obtain the relative locations between all sensors and the registration accuracy/precision conformed to the expectation of the multi-sensor CMM.

Further work should be concerned with accuracy improvement using a modified method or a compensation for the present registration method.

## Figures and Tables

**Table 1:** Circle centers and errors.

	$X_{cv}$ in 2D	$Y_{cv}$ in 2D	$Z_{cv}$ in 2D	$X_{cv}$ in 3D	$Y_{cv}$ in 3D	$Z_{cv}$ in 3D
Mean (mm)	80.0075	50.0100	70.2497	80.0075	50.0100	70.2497
SD ($10^{-3}$ mm)	0.27	0.24	>1	0.26	0.23	0.82

**Table 2:** Intersection point and errors.

	$X_{cp}$ in 3D	$Y_{cp}$ in 3D	$Z_{cp}$ in 3D
Mean (mm)	100.000	60.000	50.000
SD ($10^{-3}\;$ mm)	0.22	0.24	0.05

**Table 3:** Center of intersection points and errors.

	$X_{cc}$ in 3D	$Y_{cc}$ in 3D	$Z_{cc}$ in 3D
Mean (mm)	100.000	75.000	60.000
SD ($10^{-3}$ mm)	0.61	0.50	0.62

**Table 4:** Offsets from the VC to the TP (unit: mm).

Time of Rotation	$X_{cv}$	$Y_{cv}$	$Z_{cv}$	$X_{cp}$	$Y_{cp}$	$Z_{cp}$	PV-dx	PV-dy	PV-dz
1st	−34.9574	−91.3274	−71.6481	−34.9502	−91.3273	−71.8783	−0.0072	−0.0002	0.2302
2nd	−5.2449	−89.118	−71.6363	−5.23818	−89.1179	−71.8636	−0.0067	0.0001	0.2273
3rd	−36.5322	−15.477	−71.6455	−36.5225	−15.4784	−71.8733	−0.0098	0.0015	0.2278
SD	-	-	-	-	-	-	0.00135	0.00076	0.00125

**Table 5:** Offsets from the CC to the TP (unit: mm).

Time of Rotation	$X_{cc}$	$Y_{cc}$	$Z_{cc}$	$X_{cp}$	$Y_{cp}$	$Z_{cp}$	PC-dx	PC-dy	PC-dz
1st	−34.9393	−91.346	−72.1335	−34.9502	−91.3272	−72.1283	0.0109	−0.0188	−0.0052
2nd	−5.22586	−89.1371	−72.1209	−5.23811	−89.118	−72.1136	0.0122	−0.0191	−0.0073
3rd	−36.5102	−15.4965	−72.1286	−36.5223	−15.4784	−72.1233	0.0121	−0.0181	−0.0053
SD	-	-	-	-	-	-	0.00062	0.00045	0.00098

**Table 6:** Offsets from the CCs to the TPs with basic calculation (unit: mm).

Time of Rotation	$X_{cc}$	$Y_{cc}$	$Z_{cc}$	$X_{cp}$	$Y_{cp}$	$Z_{cp}$	PC-dx	PC-dy	PC-dz
1st	−34.9389	−91.3469	−72.1336	−34.9502	−91.3272	−72.1283	0.0112	−0.0197	−0.0053
2nd	−5.22524	−89.1362	−72.1208	−5.23811	−89.118	−72.1136	0.0129	−0.0182	−0.0072
3rd	−36.5104	−15.4973	−72.1287	−36.5223	−15.4784	−72.1233	0.0119	−0.0188	−0.0053
SD	-	-	-	-	-	-	0.00067	0.00060	0.00089

**Table 7:** Offsets between sensors with a sphere unit (unit: mm).

Time	PV-dx	PV-dy	PV-dz	PC-dx	PC-dy	PC-dz
1	−0.0062	0.0008	-	0.0012	−0.0176	−0.0088
2	−0.0067	0.0005	-	0.0011	−0.0178	−0.0090
3	−0.0063	0.0008	-	0.0014	−0.0179	−0.0074
4	−0.0065	0.0006	-	0.0011	−0.0180	−0.0088
5	−0.0061	0.0007	-	0.0014	−0.0180	−0.0082
6	−0.0066	0.0006	-	0.0013	−0.0181	−0.0090
mean	−0.0064	0.0007	-	0.0013	−0.0179	−0.0085
SD	0.00020	0.00012	-	0.00012	0.00016	0.00056
